# Evaluation of cholinesterase inhibitory and antioxidant activity of *Wedelia chinensis* and isolation of apigenin as an active compound

**DOI:** 10.1186/s12906-021-03373-4

**Published:** 2021-07-27

**Authors:** Md. Aminul Islam, Shahed Zaman, Kushal Biswas, Md. Yusuf Al-Amin, Md. Kamrul Hasan, A. H. M. K. Alam, Toshihisa Tanaka, Golam Sadik

**Affiliations:** 1 Department of Chemistry, University of Rajshahi, Rajshahi, 6205 Bangladesh; 2 Department of Pharmacy, University of Rajshahi, Rajshahi, 6205 Bangladesh; 3 Psychiatry, Graduate School of Medicine, Tsuita, Osaka, Japan

**Keywords:** *Wedelia chinensis*, Alzheimer’s disease, Cholinesterase inhibition, Antioxidant activity, Apigenin

## Abstract

**Background:**

*Wedelia chinensis* has been reported as a folk medicine for the treatment of different diseases including neurodegenerative disease. Although the plant has been studied well for diverse biological activities, the effect of this plant in neurological disorder is largely unknown. The present study was undertaken to evaluate the cholinesterase inhibitory and antioxidant potential of *W. chinensis*.

**Methods:**

The extract and fractions of the plant were evaluated for acetylcholinesterase and butyrylcholinesterase inhibitory activity by modified Ellman method. The antioxidant activity was assessed in several in vitro models/assays such as reducing power, total antioxidant capacity, total phenolic and flavonoid content, scavenging of 2,2′-diphenyl-1-picrylhydrazyl (DPPH) free radical and hydroxyl radical, and inhibition of brain lipid peroxidation. Chromatographic and spectroscopic methods were used to isolate and identify the active compound from the extract.

**Results:**

Among the fractions, aqueous fraction (AQF) and ethylacetate fraction (EAF) exhibited high inhibition against acetylcholinesterase (IC_50_: 40.02 ± 0.16 μg/ml and 57.76 ± 0.37 μg/ml) and butyrylcholinesterase (IC_50_: 31.79 ± 0.18 μg/ml and 48.41 ± 0.05 μg/ml). Similarly, the EAF and AQF had high content of phenolics and flavonoids and possess strong antioxidant activity in several antioxidant assays including DPPH and hydroxyl radical scavenging, reducing power and total antioxidant activity. They effectively inhibited the peroxidation of brain lipid in vitro with IC_50_ values of 45.20 ± 0.10 μg/ml and 25.53 ± 0.04 μg/ml, respectively. A significant correlation was observed between total flavonoids and antioxidant and cholinesterase inhibitory activity. Activity guided chromatographic separation led to the isolation of a major active compound from the EAF and its structure was elucidated as apigenin by spectral analysis.

**Conclusions:**

The potential ability of *W. chinensis* to inhibit the cholinesterase activity and peroxidation of lipids suggest that the plant might be useful for the management of AD.

**Supplementary Information:**

The online version contains supplementary material available at 10.1186/s12906-021-03373-4.

## Background

Medicinal plant has been used as folk medicine in many countries for the management of various diseases. In Bangladesh, the majority of the population depends on traditional plant-based medicines for the management of various ailments including neurodegenerative disorders [1]. There are about 1000 plant species in the country that have been recorded to have medicinal qualities, out of which 250 are regularly used in medicines [2]. It is therefore plausible that scientific evaluation of plants with high potential in traditional medicine could be useful to develop novel drugs or nutraceuticals for different disorders. *Wedelia chinensis* (Synonyms: *Wedelia calendulacea*), locally known as Bhringoraj, belongs to the family Asteraceae. It is grown in Dhaka, Mymenshingh, Tangail, Patuakhali, Barisal and sporadically in some other areas of Bangladesh. The plant is traditionally used to treat liver enlargement, jaundice and other ailments of the liver and gall bladder. It is also used in the treatment of rheumatic fever, headache, dysentery, cough, cephalalgia, diseases of skin, uterine hemorrhage and menorrhagia. The leaves of the plant are often used for dyeing grey hair, promoting hair growth and tonic [3–5]. It has been reported that the decoction of the plant is used for strengthening the nervous system and to treat multiple sclerosis [6]. The plant is indicated for many ailments in traditional Ayurvedic and Unani system of medicine. Biological investigations of this plant have shown that the plant possesses multiple pharmacological effects such as anti-cancer, anti-hepatotoxic, anti-inflammatory, anti-microbial and anti-oxidant activities [7–11]. In a neuropharmacological study, the plant exhibited a protective role in D-galactose induced neuronal cell loss and CNS depressant activity in mice [12, 13]. Phytochemical study reported the isolation of four active compounds, such as wedelolactone, indole-3-carboxylaldehyde, apigenin and luteolin, of which the latter two compounds are flavonoids [8]. These compounds are reported to have antioxidant, anti-inflammatory and neuroprotective properties [14–16]. The other species of Asteraceae family have shown a wide range of anti-inflammatory, antimicrobial, antioxidant and hepatoprotective activities [17]. Although the plant *W. chinensis* has demonstrated a magnitude of therapeutic activities, the protective effect of this plant in neurodegenerative diseases is largely unknown.

Degeneration of the central nervous system results in a variety of neurological disorders including Alzheimer’s disease (AD). AD is the most devastating neurodegenerative disorder of the elderly people and the most common cause of dementia. Cholinergic neuron, which is involved in the regulation of memory and cognition, is severely impaired in AD. The most remarkable features found in AD are cholinergic dysfunction associated with a progressive decline in neurotransmitter acetylcholine (ACh) [18]. Activity of acetylcholine in the brain is terminated by the hydrolysis of cholinesterase. Therefore, inhibition of cholinesterases has become promising therapeutic approach in AD. In addition, oxidative stress has been detected as one of the common neurotoxic pathway in neurodegenerative diseases including AD. It has been shown that Aβ protein, which is excessively generated in AD, can produce reactive oxygen species including free radical, and make an imbalance between ROS and antioxidant system leading to oxidative stress [19, 20]. Free radicals are capable of attacking most of the cellular biomolecules such as DNA, protein, and lipid. Among the oxidized molecules, increased peroxidation of lipid has been found in the brain of AD which can be determined as thiobarbituric reactive species [21, 22]. The increased peroxidation of lipid might result from the relative abundance of polyunsaturated fatty acid in the brain. Therefore, researchers have been devoted to develop an agent for the treatment of AD that would target both the cholinesterases and oxidative stress. Plants are important sources of novel drugs with diverse biological properties including antioxidant and anti-cholinesterase activities. Interest in medicinal plants has increased in recent times due to toxicity of the synthetic drugs.

*W. chinensis* has been studied earlier for antioxidant activity which were preliminary in nature [23, 24] and no cholinesterase inhibitory property of this plant has been examined yet. Therefore, this study was an attempt to evaluate the cholinesterase inhibitory and antioxidant activities of the extracts of *W. chinensis* using in vitro models and to isolate and characterize the active constituent.

## Methods

### Chemicals

Donepezil (CAS 120011–70-3), galantamine (CAS 1953-04-4), acetylthiocholine iodide (CAS 1866-15-5) and S-butyrylthiocholine iodide (CAS 1866-16-6), DPPH (2,2′-diphenyl-1-picrylhydrazyl) (CAS 1898-66-4), ammonium molybdate, Folin-Ciocalteu reagent, thiobarbituric acid (TBA) (CAS 504–17-6), tricholoroacetic acid (TCA), 2-deoxy-D-ribose (CAS 533–67-5), 5,5′-dithio-bis-(2-nitro) benzoic acid (DTNB) (CAS 69–78-3), triton X-100, aluminum chloride, potassium ferricyanide, and Tris-HCl were procured from Sigma-Aldrich, Germany. Catechin (CAS 154–23-4), ascorbic acid and gallic acid (CAS 149–91-7) were obtained from Wako Pure Chemical Company Ltd., Japan. Methanol, ethylacetate, chloroform and petroleum ether were purchased from Active Fine Chemicals Limited, Dhaka, Bangladesh. All other chemicals, unless specified, were of analytical grade.

### Animals, homogenization of brain and extraction of acetylcholinesterase

Mice were used only for collection of brain as a source of crude acetylcholinesterase enzyme. Swiss Albino mice having 5–6 weeks were purchased from the Animal House, Jahangirnagar University, Savar, Dhaka and were caged in the experimental room. A standard diet and water ad libitum were given to the mice. The study was carried out in compliance with the ARRIVE guidelines. The international ethical guidelines were followed to deal with the laboratory animals. The procedures were approved by the by the Institutional Animal, Medical Ethics, Biosafety and Biosecurity Committee (IAMEBBC) of the University of Rajshahi, Bangladesh vide reference number 255(14)/320/IAMEBBC/IBSc.

To collect brain from mice, mice were anesthetized with sodium pentobarbital (30 mg/kg; intraperitoneal injection; Taj Pharmaceuticals Ltd., India) and then sacrificed by cervical dislocation. Cervical dislocation was performed by a trained person. Brain tissues were taken quickly, washed in ice cold saline and used for preparation of acetylcholinesterase (AChE) by the method as described earlier [25, 26]. In brief, brain tissues were homogenized in Tris-saline buffer (50 mM Tris-HCl, 1.0 M NaCl, and 50 mM MgCl_2_, pH 7.4) containing 1% (wt/vol) Triton X-100 and then centrifuged at 10,000 rpm for 30 min at 4 °C to yield the crude AChE. AChE activity was determined by the method of Ellman [27]. One unit of AChE activity was defined as the number of micromole of acetylthiocholine iodide hydrolyzed per minute at 22 °C. Protein concentration in the AChE extract was determined using Lowry method with bovine serum albumin as standard [28]. The specific activity of the prepared AChE was 78 U/mg.

### Plant collection, extraction and fractionation

The whole plant was collected from the district of Natore, Bangladesh in March 2016 after obtaining permission from the owner and authenticated by Professor Dr. A.H.M. Mahbubur Rahman, Department of Botany, Rajshahi University, where a voucher specimen (accession no. 370) have been deposited. The use of plant parts in the present study complies with international, national and/or institutional guidelines.

The plant material, after washing with distilled water, was cut into small pieces, shade dried for several days and then ground to a coarse powder by grinding machine. To prepare the crude methanol extract (CME), the powder material (500 g) was extracted with methanol in a Soxhlet apparatus by hot extraction method and filtered through cotton bed followed by Whatman filter paper number 1. The filtrate was concentrated in vacuo with a rotary evaporator to obtain semisolid mass (18.5 g). The CME (10 g) was suspended in 10% methanol (200 ml) and then sequentially partitioned with petroleum ether (3 × 200 ml), chloroform (3 × 200 ml), ethylacetate (3 × 200 ml) and water (3 × 200 ml) by the method as described earlier [25, 29] to yield the corresponding petroleum ether (PEF, 3.2 g), chloroform (CLF, 2.5 g), ethylacetate (EAF, 1.4 g) and aqueous (AQF, 2.9 g) fractions. All the fractions were preserved in a refrigerator at 4 °C until further use.

### Phytochemical analysis

#### Phytochemical screening of the plant extract

Qualitative tests were performed to identify the classes of phytochemicals such as flavonoids, alkaloids, tannins, saponins, and steroids in the different fractions by the methods as described earlier [30].

#### Quantitation of total phenolic content (TPC)

Folin-Ciocalteu method was used to determine the total phenolic content of the extractives of *W. chinensis* as described earlier [31]. To a mixture of 2.5 ml of 10% Folin-Ciocalteu reagent and 2.5 ml of 7.5% sodium carbonate solution, 0.5 ml sample was added and left in the dark for 20 min at 25 °C. The absorbance of the reaction mixture was recorded by a spectrophotometer at 760 nm. A standard curve was obtained for gallic acid and the phenolic content was determined from extrapolation of this curve.

#### Quantitation of total flavonoid content (TFC)

Aluminum chloride colorimetric method was used to measure the total flavonoid content of the extracts of *W. chinensis* as described earlier [32]. To a mixture of methanol (3.0 ml), 10% AlCl_3_ (0.2 ml), 1 M potassium acetate (0.2 ml) and 5.6 ml of distilled water, 1 ml plant extract was added and left at room temperature for 30 min. The absorbance of the reaction mixture was recorded by a spectrophotometer at 420 nm. A standard curve was obtained for catechin and the flavonoid content was determined from extrapolation of this curve.

### Antioxidant activity

#### Reducing power assay

The reducing ability of the extracts of *W. chinensis* was determined by the method of Oyazu et al. (1986) [33]. To a mixture of 0.2 M potassium buffer (2.5 ml) and 1% potassium ferricyanide (2.5 ml), 1 ml plant extract (5–80 μg/ml concentration) was added and incubated at 50 °C for 20 min. Then 10% TCA solution (2.5 ml) was added to the reaction mixture and centrifuged (3000 rpm) for 10 min. Finally, 2.5 ml of solution was mixed with 2.5 ml of ultrapure water and 0.5 ml of 0.1% ferric chloride solution. The absorbance of the reaction mixture was recorded at 700 nm. A reference standard catechin was used for comparison.

#### Total antioxidant capacity assay

The antioxidant capacity of the extracts of *W. chinensis* was assessed by the method as described earlier [30]. To a mixture of sulphuric acid (0.6 M), sodium phosphate (28 mM) and ammonium molybdate (4 mM), plant extract at a concentration of 5–80 μg/ml was added and heated in a water bath at 95 °C for 90 min. After cooling to room temperature, the absorbance of the mixture was recorded at 695 nm against blank. A reference compound catechin was used for comparison.

#### DPPH radical scavenging assay

The ability of the extracts of *W. chinensis* to scavenge DPPH radical was measured by the modified method of Choi et al. (2000) [34]. A reference compound catechin was used for comparison. Methanolic solution of plant extract or reference compound (6.25–100 μg/ml concentration) was mixed with 0.135 mM of methanolic DPPH and left in dark for 30 min. The absorbance of the reaction mixture was recorded at 517 nm. The percent scavenging was calculated using the equation:

$$ \left[\left({\mathrm{A}}_{\mathrm{control}}-{\mathrm{A}}_{\mathrm{sample}}\right)/{\mathrm{A}}_{\mathrm{control}}\right]\times 100 $$

Where, A _control_ is the absorbance of control and A _sample_ is the absorbance of extract or reference compound. The percentage inhibition was plotted against the compound concentration in order to calculate the IC_50_ values.

#### Determination of hydroxyl radical scavenging activity

The capacity of the extracts of *W. chinensis* to scavenge hydroxyl radical was assessed by the modified method of Elizabeth et al. (1990) [35]. A reference compound catechin was used for comparison. Plant extract or reference compound (6.25–100 μg/ml concentration) was added to a 1 ml reaction mixture containing 2.8 mM 2-deoxy-2-ribose, 20 mM phosphate buffer (pH 7.4), 100 μM FeCl_3_, 100 μM EDTA, 1 mM H_2_O_2_ and 100 μM ascorbic acid and then incubated at 37 °C for 60 min. 0.5 ml of the reaction mixture was mixed with 1 ml of TCA (2.8%) and 1 ml of TBA (1%) and heated in a water bath at 90 °C for 15 min. After cooling to room tempaerature, the absorbance of the mixture was recorded at 532 nm in a spectrophotometer against an appropriate blank solution. The percent scavenging of hydroxyl radical was calculated as in DPPH radical scavenging assay.

#### Determination of lipid peroxidation inhibition activity

The ability of the extracts of *W. chinensis* to inhibit the peroxidation of lipid was assesses by the method as described [30]. Brain homogenate was employed for in vitro lipid peroxidation assay. Mice brain homogenates were prepared by homogenizing brain in 50 mM phosphate buffer (pH 7.4) containing 0.15 M KCl using a homogenizer and centrifuged at 10000 *g* at 4 °C for 20 min. To a mixture of brain homogenates (0.5 ml), 0.15 M KCl (1 ml) and 10 μM hydrogen peroxide (100 μl), plant extract (6.25–100 μg/ml concentration) was added and incubated at 37 °C for 30 min. A solution of 2 ml of HCl (0.25 N) containing TCA (15%), TBA (0.38%), and BHT (5%) was added to the reaction mixture and heated in a water bath at 80 °C for 60 min. After cooling to room temperature, the mixture was centrifuged to separate the supernatant and then the absorbance was measured at 532 nm by spectrophotometer. The percent inhibition of lipid peroxidation was determined as in DPPH radical scavenging assay. A reference standard catechin was used for comparison.

### Cholinesterase inhibitory activities

The assessment of acetylcholinesterase (AChE) and butyrylcholinesterase (BChE) inhibiting activities were performed by the colorimetric method of Ellman et al. (1961) [27]. Crude AChE enzyme was prepared from mice brain as mentioned above and BChE enzyme was prepared from human blood according to the method as described earlier [25, 30]. The acetylthiocholine iodide and S-butyrylthiochoilne iodide were used as substrates for investigation of AChE and BChE assays, respectively. The hydrolysis of acetylthiocholine and S-butyrylthiocholine were determined spectrophotometrically. Plant extract or reference compound (12.5–200 μg/ml concentration) was added in an enzyme solution and incubated at 37 °C for 15 min for interaction. This was followed by the addition of a 50 mM sodium phosphate buffer (pH 8.0) containing 0.5 mM acetylthiocholine and 1 mM DTNB and immediately the absorbance of the solution was recorded against a blank solution. All the experiments were taken in triplicate. For comparison, a reference compound donepezil was used for AChE activity and galantamine was used for BChE activity. The percent inhibition of cholinesterase activity was computed using the equation:

$$ \left[\left({\mathrm{A}}_{\mathrm{control}}-{\mathrm{A}}_{\mathrm{sample}}\right)/{\mathrm{A}}_{\mathrm{control}}\right]\times 100 $$

Where, A _control_ is the absorbance of control and A _sample_ is the absorbance of extract or reference compound. IC_50_ value could be calculated from the dose response curve obtained by plotting the percent inhibition values against test concentrations of each extract or compound.

### Isolation and characterization of an active compound from the bioactive extract

The EAF (5.6 g) of *W. chinensis* was subjected to column chromatography using silica gel 60 (Merck, Germany) as a stationery phase in an open column and sequentially eluted with *n*-hexane, dichloromethane and methanol stepwise gradient to yield five major subfractions (F1 to F5). Fraction F2 with potent AChE and BChE inhibitory activity was purified on silica gel GF_254_ by preparative thin layer chromatography with *n*-hexane-acetone (6:4) as the mobile phase to obtain the pure compound **1** (18 mg).

^1^H- and ^13^ C-NMR spectra of the compound **1** was recorded in DMSO-*d*_6_ on a Jeol-Ex 400 MHz and FT-NMR 100 MHz spectrometers. The chemical structure of the compound **1** was confirmed by comparing its spectral data with the reported values in the literature [36].

### Statistical analysis

All experiments were carried out in triplicate. The results were expressed as mean ± SD. Graph Pad Prism (version 8.0.1) and Microsoft Excel 2010 were used for the statistical and graphical evaluations. T-test was employed to estimate the statistical significance (*P*-value **<** 0.05) between the average values. IC_50_ values of different fractions/extractives were calculated using non-linear regression (Dose-Response -- Inhibition equation; log_10_ (inhibitor) vs. normalized response -- variable slope) in Graph Pad Prism - 8.0.1. Correlation study was performed using Pearson correlation test.

## Results

### Phytochemical analysis

A preliminary phytochemical analysis conducted on the CME revealed that the plant contains tannins, phenolics and flavonoids, alkaloids, phytosterols and saponins. Qualitative analysis of the four fractions showed that they all contained phenolics and flavonoids, but higher amounts were found in the EAF and AQF (Supplementary Table S1).

Assays for total phenolic and flavonoid content of the extractives revealed that EAF contained the highest content of phenolics (97.28 ± 0.49 mg GAE/g dried extract) followed by CME (80.00 ± 0.62 mg GAE/g dried extract), AQF (61.05 ± 0.21 mg GAE/g dried extract), CLF and PEF (Table 1; Supplementary Table S2 and S3). Whereas, AQF contained the highest content of flavonoids (175.78 ± 0.69 mg CE/g dried extract), followed by CME (174.02 ± 1.01 mg CE/g dried extract), EAF (144.35 ± 0.51 mg CE/g dried extract), CLF and PEF.

**Table 1:** Total phenolic and flavonoid contents and antioxidant activity of the extract and fractions of *Wedelia chinensis*

Sample	TPC (mg GAE/g dried extract)	TFC (mg CE/g dried extract)	DPPH IC_**50**_ (μg/mL)	OH IC_**50**_ (μg/mL)	RP (absorbance at 80 μg/mL)	TAC (absorbance at 80 μg/mL)	LPI IC_**50**_ (μg/mL)
**CME**	80.00 ± 0.62^b^	174.02 ± 1.01^b^	10.13 ± 0.44^d^	42.59 ± 0.51^d^	1.783 ± 0.03^d^	0.456 ± 0.008^d^	47.18 ± 1.07^d^
**PEF**	7.34 ± 0.16^e^	2.68 ± 0.23^e^	27.46 ± 0.52^f^	160.33 ± 2.37^f^	0.942 ± 0.01^f^	0.347 ± 0.005^f^	416.20 ± 1.51^f^
**CLF**	33.52 ± 0.20^d^	33.46 ± 0.34^d^	21.53 ± 0.46^e^	105.47 ± 2.01^e^	1.383 ± 0.02^e^	0.365 ± 0.005^e^	222.60 ± 3.36^e^
**EAF**	97.28 ± 0.49^a^	144.35 ± 0.51^c^	4.78 ± 0.01^a^	28.21 ± 0.39^c^	2.245 ± 0.06^c^	0.529 ± 0.007^b^	45.20 ± 0.10^c^
**AQF**	61.05 ± 0.21^c^	175.78 ± 0.69^a^	8.60 ± 0.04^c^	9.65 ± 0.25^a^	2.542 ± 0.05^a^	0.556 ± 0.008^a^	25.53 ± 0.04^a^
**CAT**	–	–	5.14 ± 0.10^b^	14.89 ± 0.25^b^	2.451 ± 0.07^b^	0.489 ± 0.004^c^	30.91 ± 0.20^b^

PEF, petroleum ether fraction; CLF, chloroform fraction; EAF, ethylacetate fraction; AQF, aqueous fraction; CAT, catechin. TPC: Total phenolic content, TFC: Total flavonoid content, OH: Hydroxyl radical scavenging, RP: Reducing power, TAC: Total antioxidant capacity and LPI: Lipid peroxidation inhibition. Means in each column with different subscript letters (a b, c, d, e, f) differ significantly (*P* < 0.05)

### Cholinesterase inhibitory activity

**Fig. 1:**
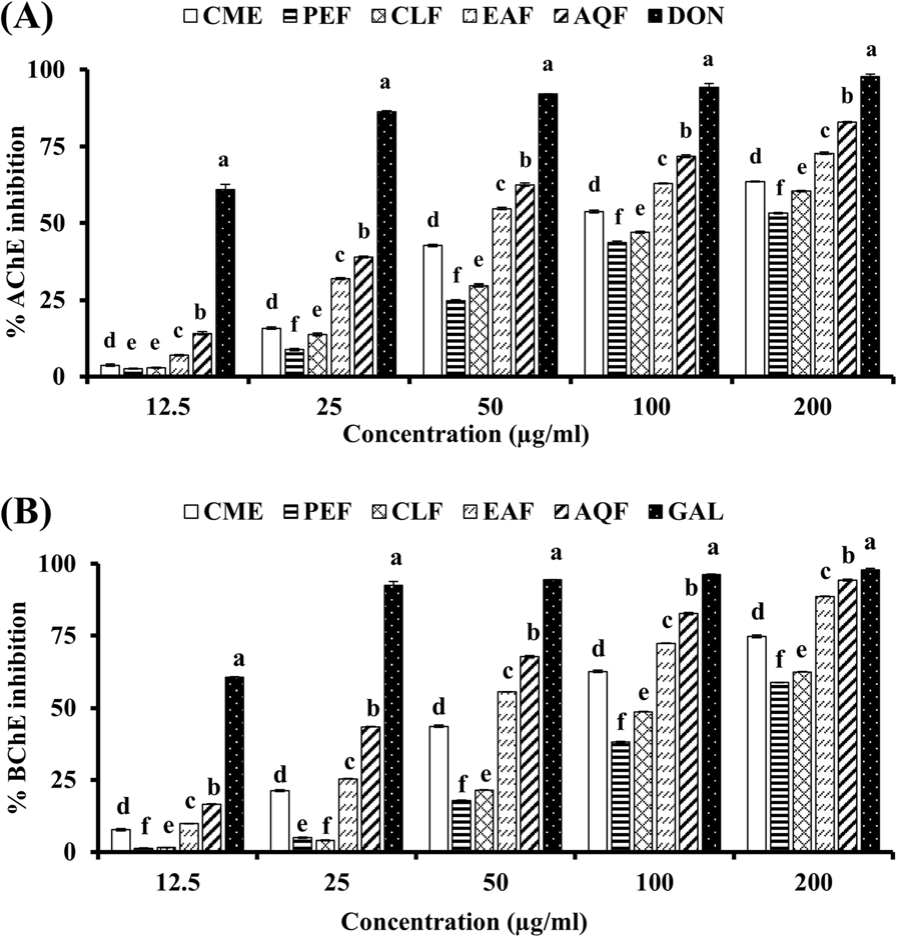
Cholinesterase inhibitory activities of the extract and fractions of *W. chinensis*. (**A**) Inhibition of acetylcholinesterase (AChE) by extract and fractions of *W. chinensis* and standard donepezil. IC_50_ (μg/ml): CME, 93.64 ± 0.28; PEF, 152.60 ± 1.14; CLF, 121.97 ± 0.74; EAF, 57.76 ± 0.37; AQF, 40.02 ± 0.16; DON, 9.21 ± 0.45. (**B**) Inhibition of butyrylcholinesterase (BChE) by extract and fractions of *W. chinensis* and standard galantamine. IC_50_ (μg/ml): CME, 69.09 ± 0.44; PEF, 148.87 ± 0.50; CLF, 122.50 ± 0.20; EAF, 48.41 ± 0.05; AQF, 31.79 ± 0.18; GAL, 10.62 ± 0.19. Results are expressed as mean ± SD (*n* = 3). Means with different letters (a-f) differ significantly (*P* < 0.05). CME, crude methanolic extract; PEF, petroleum ether fraction; CLF, chloroform fraction; EAF, ethylacetate fraction; AQF, aqueous fraction; DON, Donepezil; GAL, Galantamine

The CME and its fractions were evaluated for AChE inhibition at different concentration using the widely used Ellman method [27]. The percent inhibition of AChE by the extractives has been presented in Fig. 1A. Donepezil was used as the reference AChE inhibitor in this study that showed an IC_50_ of 9.21 ± 0.45 μg/ml. All the test extract and fractions exerted dose dependent inhibition of AChE enzyme. The IC_50_ of CME was found to be 93.64 ± 0.28 μg/ml. Among the fractions, high activity was found in AQF and EAF with IC_50_ values of 40.02 ± 0.16 μg/ml and 57.76 ± 0.37 μg/ml, respectively. CLF and PEF showed less activity with IC_50_ of 121.97 ± 0.74 μg/ml and 152.60 ± 1.14 μg/ml, respectively (Fig. 1A, Table 1). Similarly, in BChE inhibitory assay, the CME showed good activity with IC_50_ of 69.09 ± 0.44 μg/ml. AQF and EAF exhibited high inhibitory activity with IC_50_ of 31.79 ± 0.18 μg/ml and 48.41 ± 0.05 μg/ml, respectively (Fig. 1B, Table 1). The IC_50_ of CLF and PEF were 122.50 ± 0.20 μg/ml and 148.87 ± 0.50 μg/ml. Taken together, the AQF and EAF possess appreciable activity against both AChE and BChE enzymes.

### Antioxidant activity

The antioxidant activity of the extractives of *W. chinensis* were assessed by using several in vitro models such as DPPH and hydroxyl free radicals scavenging, reducing power and total antioxidant activity.

**Fig. 2:**
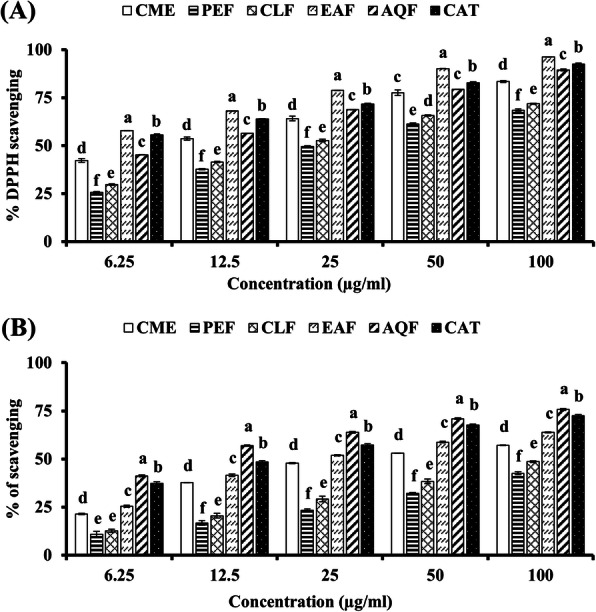
Radical scavenging activities of extract and fractions of *W. chinensis*. (**A**) DPPH radical scavenging activities of extract and fractions of *W. chinensis* and standard catechin. (**B**) Hydroxyl radical scavenging activities of extract and fractions of *W. chinensis* and standard catechin. Means with different letters (a-f) differ significantly (*P* < 0.05). CME, crude methanolic extract; PEF, petroleum ether fraction; CLF, chloroform fraction; EAF, ethylacetate fraction; AQF, aqueous fraction; CAT, catechin

DPPH is a stable free radical which is widely used for evaluation of scavenging activity of the antioxidant. The percent scavenging of DPPH free radical by different concentration of extract has been shown in Fig. 2A and Table 1. Catechin (CAT) was used as the reference antioxidant that showed an IC_50_ of 5.14 ± 0.10 μg/ml. The IC_50_ values of CME, EAF, AQF, CLF and PEF were 10.13 ± 0.44, 4.78 ± 0.01, 8.60 ± 0.04, 21.53 ± 0.46 and 27.46 ± 0.52 μg/ml, respectively, indicating that the EAF possesses the highest radical scavenging activity followed by AQF. EAF was found to be more potent than that of the standard catechin whose IC_50_ was 5.14 ± 0.10 μg/ml. The CLF and PEF had relatively lower DPPH radical scavenging activity.

Hydroxyl radical is the most harmful radical among the radicals generated in the biological system. Hydroxyl radicals were generated in vitro in Fenton reaction and the ability of the extractives to scavenge the radicals was determined (Fig. 2B, Table 1). AQF was found to possess the highest scavenging activity followed by EAF with IC_50_ values of 9.65 ± 0.25 μg/ml and 28.21 ± 0.39 μg/ml, respectively. It was noted that AQF had higher scavenging activity than that of the reference antioxidant catechin which showed an IC_50_ of 14.89 ± 0.25 μg/ml. The IC_50_ for CME was found to be 42.59 ± 0.51 μg/ml. PEF and CLF had relatively lower activity with IC_50_ of 160.33 ± 2.37 and 105.47 ± 2.01 μg/ml, respectively.

**Fig. 3:**
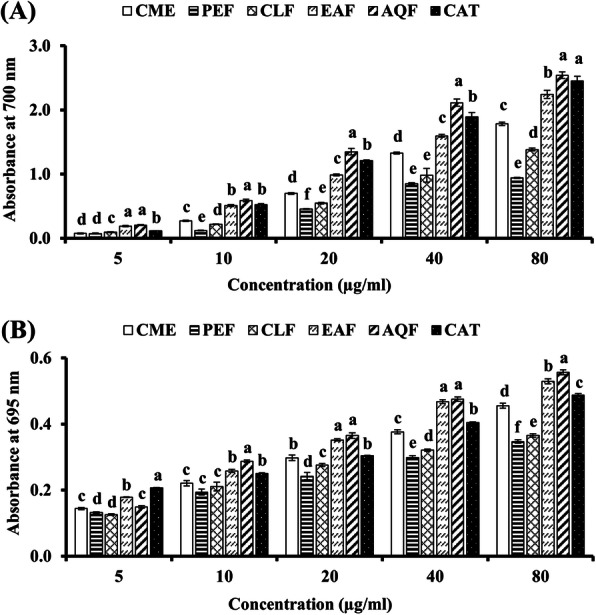
Reducing power and total antioxidant activities of extract and fractions of *W. chinensis*. (**A**) Reducing power of extract and fractions of *W. chinensis* and standard catechin. (**B**) Total antioxidant capacity of extract and fractions of *W. chinensis* and standard catechin. Results are expressed as mean ± SD (*n* = 3). Means with different letters (a-f) differ significantly (*P* < 0.05). CME, crude methanolic extract; PEF, petroleum ether fraction; CLF, chloroform fraction; EAF, ethylacetate fraction; AQF, aqueous fraction; CAT, catechin

Reducing power assay was used for assessing the reducing ability of the CME and its fractions and the result has been shown in the Fig. 3A and Table 1. All the extract and fractions were found to possess the reducing activity and the activity was increased with the increase of the concentration of the extract. At high concentration of 80 μg/ml, the absorbance of CME, AQF, EAF, CLF, PEF and CAT were 1.783 ± 0.03, 2.542 ± 0.05, 2.245 ± 0.06, 1.383 ± 0.02, 0.942 ± 0.01 and 2.451 ± 0.07, indicating that AQF has the highest activity followed by CAT, EAF, CME, CLF and PEF. Notably, the activity of AQF was found to be higher than that of the standard antioxidant catechin.

The total antioxidant activity of the extractives was assessed based on their capacity to reduce Mo (VI) to Mo (V) and the result has been shown in the Fig. 3B and Table 1. Similar to reducing power, AQF and EAF exhibited high total antioxidant activity among the extractives. At high concentration of 80 μg/ml, CME, AQF, EAF, CLF, PEF and CAT gave an absorbance of 0.456 ± 0.008, 0.556 ± 0.008, 0.529 ± 0.007, 0.365 ± 0.005, 0.347 ± 0.005 and 0.489 ± 0.004, respectively. Interestingly, the total antioxidant activity of AQF and EAF were found to be greater than that of the standard antioxidant catechin.

**Fig. 4:**
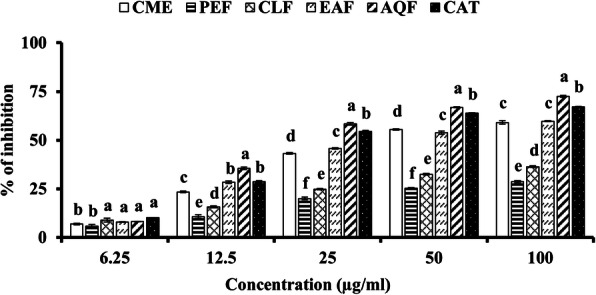
Lipid peroxidation inhibitory activity of extract and fractions of *W. chinensis* and the standard catechin. Results are expressed as mean ± SD (*n* = 3). Means with different letters (a-f) differ significantly (*P* < 0.05). CME, crude methanolic extract; PEF, petroleum ether fraction; CLF, chloroform fraction; EAF, ethylacetate fraction; AQF, aqueous fraction; CAT, catechin

Oxidation of lipid by free radicals results in lipid peroxidation. In this study, lipid peroxidation of the mouse brain homogenate was induced by hydrogen peroxide and the effect of the different extractives of *W. chinensis* in the inhibition of lipid peroxidation were assessed via thiobarbituric acid reactive species (TBARS). As shown in the Fig. 4 and Table 1, incubation of mouse brain homogenate with hydrogen peroxide caused a significant increase of lipid peroxidation. All the extracts inhibited lipid peroxidation in a dose dependent manner. Among the extractives screened, AQF and EAF exhibited high inhibitory activity with IC_50_ values of 25.53 ± 0.04 μg/ml and 45.20 ± 0.10 μg/ml, respectively. The IC_50_ of CME was 47.18 ± 1.07 μg/ml. The CLF and PEF had little activity with IC_50_ of 222.60 ± 3.36 and 416.20 ± 1.51 μg/ml, respectively. These results suggest that the AQF and the EAF can effectively inhibit the peroxidation lipid caused by free radicals*.*

### Correlation between total phenolic and flavonoid content and the acetylcholinesterase inhibitory and antioxidant activities

Phenolics and flavonoids have been reported to be associated with the antioxidant activity [37, 38]. Since AQF and EAF contained a large amount of phenolics and flavonoids and exhibited high cholinesterase inhibitory and antioxidant activities, we therefore tested their correlations by Pearson’s correlation analysis and the result has been given in the Table 2. The content of total flavonoids showed a statistically significant correlation with DPPH radical scavenging activity (R^2^ = 0.8728, *p* < 0.05), reducing activity (R^2^ = 0.7811, *p* < 0.05), total antioxidant activity (R^2^ = 0.8035, *p* < 0.05), hydroxyl radical scavenging (R^2^ = 0.9165, *p* < 0.05), lipid peroxidation inhibition (R^2^ = 0.9042, *p* < 0.05), AChE (R^2^ = 0.7748, *p* < 0.05) and BChE (R^2^ = 0.9036, *p* < 0.05) inhibitory activities. Whereas the content of phenolics showed a significant correlation with DPPH radical scavenging activity (R^2^ = 0.9263, *p* < 0.01), lipid peroxidation inhibition (R^2^ = 0.8263, *p* < 0.05) and good correlation with other antioxidant and cholinesterase activities (R^2^ = 0.62–0.77).

**Table 2:** Correlation of total phenolic and flavonoid contents with cholinesterase inhibition and antioxidant activities

Assays	R^**2**^ values	
	Total phenolic content	Total flavonoid content
Acetylcholinesterase inhibition	0.6325	0.7748*
Butyrylcholinesterase inhibition	0.7284	0.9036*
DPPH radical scavenging	0.9263*	0.8728*
Reducing power	0.6180	0.7811*
Total Antioxidant Capacity	0.6448	0.8035*
Hydroxyl radical scavenging	0.7711	0.9165*
Lipid peroxidation inhibition	0.8263*	0.9042*

* indicates statistical significance (*P <* 0.05)

### Activity guided isolation of an active compound

**Fig. 5:**
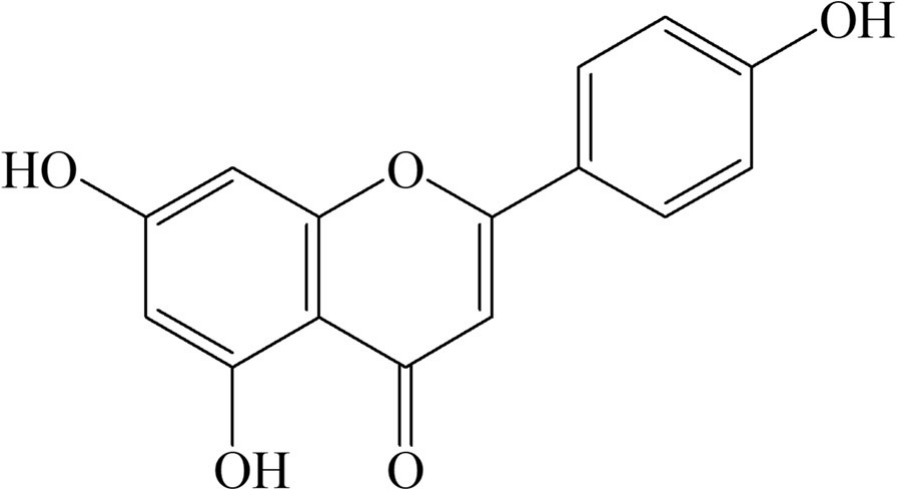
Chemical structure of the isolated compound **1**

Due to potential bioactivity, the EAF was investigated further to isolate and identify the active compounds. Activity guided chromatographic separation resulted in the isolation of a major compound **1** from the EAF and the structure of the compound **1** was established as apigenin by direct comparison of its ^1^H- and ^13^C- NMR spectral data with previously reported values (Table 3, Fig. 5 and supplementary Fig. S1 & S2) [36]. The compound exhibited inhibition of AChE and BChE enzymes as well as antioxidant activity (Table 4), which are in accordance with the previous results [39–41]. Further studies are required to isolate and identify the active compounds present in the AQF.

**Table 3:** ^1^H NMR and ^13^C NMR data (δ in ppm and *J* in Hz) of compound **1**

H/C	^**1**^H NMR	^**13**^C NMR
2	Q	157.96
3 (CH)	6.77 (1H, s)	103.51
4	Q	182.13
4a	Q	105.02
5	Q	161.43
6 (CH)	6.18 (1H, d, *J* = 2.0 Hz)	99.99
7	Q	165.25
8 (CH)	6.47 (1H, d, *J* = 2.0 Hz)	95.00
8a	Q	164.26
1’	Q	122.51
2′ (CH)	7.92 (1H, d, *J* = 8.5 Hz)	129.00
3′ (CH)	6.93 (1H, d, *J* = 8.5 Hz)	116.40
4’	Q	161.21
5′ (CH)	6.93 (1H, d, *J* = 8.5 Hz)	116.33
6′ (CH)	7.92 (1H, d, *J* = 8.5 Hz)	128.75

**Table 4:** Cholinesterase inhibitory and antioxidant activity of compound **1**

Compounds	IC_**50**_ (μM)		
	AChE	BChE	DPPH
Compound **1**	38.47 ± 0.32	36.33 ± 0.38	56.12 ± 3.14
Donepezil^a^	8.36 ± 0.68	–	–
Galantamine^a^	–	10.28 ± 0.97	–
Catechin^a^	–	–	4.43 ± 0.37

Results are expressed as mean ± SD (*n* = 3)

^a^Reference compounds used in these assays

## Discussion

Alzheimer’s disease is a progressively developing neurodegenerative disorder of the elderly people and the most common cause of dementia. Until now there is no effective treatment for AD. Approximately 50 million people are affected worldwide in AD which will triple by 2050 [42]. The problem is much more pronounced in the developing countries [43]. Due to the growing population and extended lifespan, AD has become a serious health concern in the elderly people. To date, only three cholinesterase inhibitors namely donepezil, rivastigmine, galantamine and one partial NMDA receptor antagonist memantine are the only approved drugs by the Food and Drug Administration (FDA) to treat AD [44]. These drugs offer the symptomatic relief of the disease and only slow the progression modestly, but does not stop the progression of AD [45]. The handful of drugs and the limitations of their use led us to develop new drugs. Plant has already proved to be an important source of different classes of drugs and new candidate drugs for AD has been developed. *W. chinensis* is a folk medicinal herb with multiple biological activities [7–11]. Herein, we report for the first time the cholinesterase inhibitory and antioxidant activities of the extractives of *W. chinensis* in vitro and isolation of apigenin as the major compound contributing to the activity.

Plants contain different classes of phytochemicals that contribute to the biological activity. In this study, qualitative analysis showed the presence of alkaloids, tannins, sterols, phenolics and flavonoids in the CME which were differentially distributed in the four solvent fractions (Supplementary Table S-1). Interestingly, phenolics and flavonoids were found to be present in each fraction, but high concentration was found in the EAF and AQF. Phenolics and flavonoids are an important class of secondary metabolites that are ubiquitous in plants and display important biological activities. They are known as natural antioxidants due to the ability to scavenge free radical by donating electron or hydrogen. Quantitative analysis of phenolic and flavonoid revealed a large content of phenolics (80.00 ± 0.62 mg GAE/g dried extract) and flavonoids (174.02 ± 1.01 mg CE/g dried extract) in the CME. Among the fractions of CME, high content of phenolics was found in the EAF (97.28 ± 0.49 mg GAE/g dried extract) followed by AQF (61.05 ± 0.21 mg GAE/g dried extract. Whereas the high content of flavonoid was observed in the AQF (175.78 ± 0.69 mg CE/g dried extract) followed by EAF (144.35 ± 0.51 mg CE/g dried extract) (Table 1; Supplementary Table S2 and S3). The presence of large amount of phenolics and flavonoids in the EAF and AQF indicated that they might have role in biological activity.

Inhibition of cholinesterase is still the promising therapeutic strategy for development of drugs for AD. Inhibitors of AChE increase the concentration of acetylcholine at the synapse, augment the cholinergic neurotransmission and improve the memory and cognition in animal [18]. Medicinal plants contain a diverse chemical compounds having cholinesterase inhibiting properties and currently used in the treatment of AD [46, 47]. Interest in natural AChE inhibitor has increased due to less toxic effects of the natural compounds. In this study, we report for the first time the AChE inhibitory activity of the CME of *W. chinensis* (Fig. 1A). The IC_50_ value of CME was found to be 93.64 ± 0.28 μg/ml. A large number of plants used in traditional medicine to enhance memory have been investigated, but only a few of them have been found to possess an acceptable level of AChE inhibitory capacity [48, 49]. In comparison with those plants, *W. chinensis* extract appeared to be a better AChE inhibitor. When the fractions were evaluated, high inhibition was found in the AQF and EAF with the IC_50_ values of 40.02 ± 0.16 μg/ml and 57.76 ± 0.37 μg/ml, respectively, indicating the polarity of the active compounds. Pearson’s correlation showed a significant association of total flavonoid content with the AChE inhibitory activity (R^2^ = 0.7748, *p* < 0.05) (Table 2). These results suggest that the extract of *W. chinensis* and its fractions possess an appreciable AChE inhibitory activity which might be attributed to the flavonoid compounds.

After AChE, BChE is another drug target of choice for AD. BChE is a cholinesterase involved in the catalysis of acetylcholine and plays a co-regulatory role in the cholinergic neurotransmission that accounts for 20% of cholinesterase activity [50]. The increased activity of BChE in the late stage of pathogenesis suggests its involvement in AD. There are reports that dual inhibition of AChE and BChE provide a better outcome in AD [51]. Of the three approved AChE inhibitor, rivastigmine exhibits dual activity. In this investigation, the crude methanol extract of *W. chinensis* was found to exert inhibition of BChE with an IC_50_ value of 69.09 ± 0.44 μg/ml (Fig. 1B). This result suggests that the extract has good BChE inhibitory activity and exhibits similar specificity for BChE and AChE. Among the fractions of CME, EAF and AQF showed high inhibition against BChE with IC_50_ values of 48.41 ± 0.05 μg/ml and 31.79 ± 0.18 μg/ml, respectively, indicating the polar nature of the active compounds. Pearson’s correlation showed a significant association (R^2^ = 0.9036, *p* < 0.05) of flavonoid content with BChE inhibitory activity (Table 2). This suggests that the flavonoids may be involved in the inhibition of BChE. Taken together, these results suggest a dual role of *W. chinensis* and hold promise for treating AD.

Oxidative stress plays a crucial role in the development of AD. Oxidative stress results from the excessive production of free radicals by amyloid beta-protein, a major culprit according to amyloid cascade hypothesis [19, 20]. Although a number of biomolecules are affected due to oxidative stress, lipids in neuronal membrane are most vulnerable that lead to neuronal dysfunction and death. Lipid peroxidation is thus considered as an important marker for oxidative stress in AD [21, 22]. In this study, we found the antioxidant activity of the CME and its fractions in all antioxidant assays. The activity of CME was found to be better when compared with the other medicinally important plants [49]. In DPPH radical scavenging, which is a stable synthetic radical, EAF showed the highest activity and the activity appeared to be higher than that of the reference antioxidant catechin (Fig. 2A). In hydroxyl radical scavenging, which is relevant to biological system, AQF showed the highest activity and found to be more potent than the standard antioxidant catechin (Fig. 2B). These results suggest that EAF and AQF are strong radical scavengers in terms of hydrogen donating abilities. Similarly, in reducing power and total antioxidant activity, which indicate the hydrogen and proton donating abilities, the AQF and EAF showed marked activity (Fig. 3A and B). Notably, the activities of both AQF and EAF were found to be greater or close to the activity of the standard antioxidant catechin. The antioxidant activity of the crude extract and its fractions was reflected in the inhibition of peroxidation of brain lipid from mouse (Fig. 4). In earlier investigations, the antioxidant activities of the crude methanol extract and the flavonoid rich ethylacetate fraction of the plant have been reported by Banu and Nagarajan [23] and Pavithra et al. [24] which are consistent with our results. The authors did not quantify the phytochemicals including phenolics and flavonoids in their study, which is important to understand their relationship with the activity. In our study, we show by Pearson’s correlation a strong association of total flavonoid content with DPPH radical scavenging, reducing power, total antioxidant activity, hydroxyl radical scavenging and lipid peroxidation inhibition (Table 2). While a significant correlation was observed between total phenolic content and DPPH radical scavenging activity, and lipid peroxidation inhibition and a moderate association was found between total phenolic content with hydroxyl radical scavenging activity. These results indicated a strong association of flavonoids with both the cholinesterase inhibitory and antioxidant activities.

To gain insights into the compounds responsible for activity, we explored the bioactive compounds in EAF by bioassay-guided chromatography. A major active compound was isolated from EAF and its structure was established as apigenin by ^1^H- and ^13^C- NMR spectral studies (Table 3, Fig. 5 and supplementary Fig. S1 & S2) [35]. The compound exerted inhibition of AChE and BChE enzymes and displayed antioxidant activity (Table 4). The activities of the compound were similar to that of the activities reported previously [39–41]. The molecular interaction of apigenin with AChE and BChE was supported by several molecular docking studies [40, 52, 53]. Apigenin is one of the most naturally occurring flavonoids that are found in edible and medicinal plants. The compound has been reported earlier to inhibit Aβ aggregation, and neuroinflammation involved in AD [54]. Apigenin can be absorbed in the intestine and cross the blood brain barrier, suggesting that it can be used as a therapeutic agent in the neurodegenerative pathologies including AD [14]. In the present study, the isolation of apigenin from *W. chinensis* having both cholinesterase inhibitory and antioxidant properties, which according to the previous report, can be useful to prevent or slow down the progression of AD. Taken together, all these evidences suggest that *W. chinensis* has neuroprotective potential to prevent or slow down the progression of AD.

## Conclusion

Our results demonstrated that *Wedelia chinensis* exerts substantial inhibition of cholinesterase activity and antioxidant properties, which could be useful for the management of Alzheimer’s disease. Apigenin was identified as a major compound that contributes to the inhibitory activities. To the best our knowledge, our studies are the first to report the cholinesterase inhibition and antioxidant properties of this plant. The present findings warrant further evaluation of this plant in in vivo animal models.

## Supplementary Information

**Additional file 1 Table S1.** Qualitative phytochemical screening of the solvent fractions from the extract of *Wedelia chinensis*. **Table S2.** Determination of total phenolic content of the extract and fractions of *W. chinensis.* **Table S3.** Determination of total flavonoid content of the extract and fractions of *W. chinensis.* **Fig. S1** ^1^H NMR (400 MHz, DMSO*-d*_6_) spectrum of compound 1. **Fig. S2** ^13^C NMR (100 MHz, DMSO-*d*_6_) spectrum of compound 1.

## Data Availability

The datasets used and/or analyzed during the current study are available from the corresponding author on reasonable request.

## Abbreviations

AD: Alzheimers’s disease
AChE: Acetylcholinesterase
BChE: Butyrylcholinesterase
DPPH: 2,2′-diphenyl-1-picrylhydrazyl
TPC: Total phenolic content
TFC: Total flavonoid content
CME: Crude methanolic extract
PEF: Petroleum ether fraction
CLF: Chloroform fraction
EAF: Ethylacetate fraction
AQF: Aqueous fraction
DON: Donepezil
GAL: Galantamine
CAT: Catechin
