# Supplementary material for: Evaluation of cholinesterase inhibitory and antioxidant activity of Wedelia chinensis and isolation of apigenin as an active compound
Source: BMC Complement Med Ther. 2021 Jul 27;21:204. doi: 10.1186/s12906-021-03373-4 (PMC8317308; doi:10.1186/s12906-021-03373-4)
Supplement: Supplementary file 1 — Additional file 1 Table S1. Qualitative phytochemical screening of the solvent fractions from the extract of Wedelia chinensis. Table S2. Determination of total phenolic content of the extract and fractions of W. chinensis. Table S3. Determination of total flavonoid content of the extract and fractions of W. chinensis. Fig. S1 1H NMR (400 MHz, DMSO-d6) spectrum of compound 1. Fig. S2 13C NMR (100 MHz, DMSO-d6) spectrum of compound 1. [file 12906_2021_3373_MOESM1_ESM.doc]

**Supplementary Information**

**Evaluation of cholinesterase inhibitory and antioxidant activity of *Wedelia chinensis* and isolation of apigenin as an active compound**

Md. Aminul Islam^1^, Shahed Zaman^1^, Kushal Biswas^2^, Md. Yusuf Al-Amin^2^, Md. Kamrul Hasan^2^, A.H.M.K. Alam^2^, Toshihisa Tanaka^3^ and Golam Sadik^2^*

^1^Department of Chemistry, University of Rajshahi, Rajshahi-6205, Bangladesh

^2^Department of Pharmacy, University of Rajshahi, Rajshahi-6205, Bangladesh

^3^Psychiatry, Graduate School of Medicine, Osaka, Tsuita, Osaka, Japan

*Corresponding author:

Dr. Md. Golam Sadik, Professor, Department of Pharmacy, Rajshahi University, Rajshahi-6250, Bangladesh, E-mail: [gsadik2@ru.ac.bd](mailto:gsadik2@ru.ac.bd)

**Table S1.** Qualitative phytochemical screening of the solvent fractions from the extract of *Wedelia chinensis*.

|  | **PEF** | **CLF** | **EAF** | **AQF** |
| --- | --- | --- | --- | --- |
| Tannins | - | - | - | ++ |
| Flavonoids | + | ++ | +++ | +++ |
| Phenolic compounds | + | ++ | +++ | +++ |
| Alkaloids | - | ++ | + | + |
| Saponins | - | - | - | + |
| Phytosterols | ++ | + | - | - |

Here, + = Present in mild amount, ++ = Present in moderate amount, +++ = Present in large amount, - = Absence. PEF, petroleum ether fraction; CLF, chloroform fraction; EAF, ethylacetate fraction; AQF, aqueous fraction.

**Table S2.** Determination of total phenolic content of the extract and fractions of *W. chinensis*

| **Sample** | **Conc (µg/mL)** | **No. of trials** | **Abs** | **GAE (mg/gm)** | | |
| --- | --- | --- | --- | --- | --- | --- |
|  |  |  |  |  | **Avg** | **SD** |
| **CME** | 50 | A | 0.574 | 80.20 |  |  |
|  | 50 | B | 0.568 | 79.30 | **80.00** | **0.62** |
|  | 50 | C | 0.576 | 80.49 |  |  |
| **CHF** | 50 | A | 0.243 | 33.69 |  |  |
|  | 50 | B | 0.242 | 33.57 | **33.52** | **0.20** |
|  | 50 | C | 0.240 | 33.30 |  |  |
| **EAF** | 50 | A | 0.696 | 97.33 |  |  |
|  | 50 | B | 0.699 | 97.75 | **97.28** | **0.49** |
|  | 50 | C | 0.692 | 96.77 |  |  |
| **AQF** | 50 | A | 0.439 | 61.26 |  |  |
|  | 50 | B | 0.438 | 61.04 | **61.05** | **0.21** |
|  | 50 | C | 0.436 | 60.84 |  |  |
| **PEF** | 50 | A | 0.056 | 7.50 |  |  |
|  | 50 | B | 0.054 | 7.19 | **7.34** | **0.16** |
|  | 50 | C | 0.055 | 7.32 |  |  |

Here, CME, crude methanolic extract; PEF, petroleum ether fraction; CLF, chloroform fraction; EAF, ethylacetate fraction; AQF, aqueous fraction; GAE, gallic acid equivalent.

**Table S3.** Determination of total flavonoid content of the extract and fractions of *W. chinensis*

| **Sample** | **Conc (µg/mL)** | **No. of trials** | **Abs** | **CE (mg/gm)** | | |
| --- | --- | --- | --- | --- | --- | --- |
|  |  |  |  |  | **Avg** | **SD** |
| **CME** | 250 | A | 0.551 | 174.97 |  |  |
|  | 250 | B | 0.545 | 172.95 | **174.02** | **1.01** |
|  | 250 | C | 0.549 | 174.14 |  |  |
| **CHF** | 250 | A | 0.148 | 33.77 |  |  |
|  | 250 | B | 0.148 | 33.51 | **33.46** | **0.34** |
|  | 250 | C | 0.147 | 33.10 |  |  |
| **EAF** | 250 | A | 0.465 | 144.77 |  |  |
|  | 250 | B | 0.462 | 143.79 | **144.35** | **0.51** |
|  | 250 | C | 0.464 | 144.49 |  |  |
| **AQF** | 250 | A | 0.555 | 176.49 |  |  |
|  | 250 | B | 0.553 | 175.75 | **175.78** | **0.69** |
|  | 250 | C | 0.551 | 175.11 |  |  |
| **PEF** | 250 | A | 0.059 | 2.46 |  |  |
|  | 250 | B | 0.060 | 2.66 | **2.68** | **0.23** |
|  | 250 | C | 0.061 | 2.91 |  |  |

Here, CME, crude methanolic extract; PEF, petroleum ether fraction; CLF, chloroform fraction; EAF, ethylacetate fraction; AQF, aqueous fraction; CE, catechin equivalent.


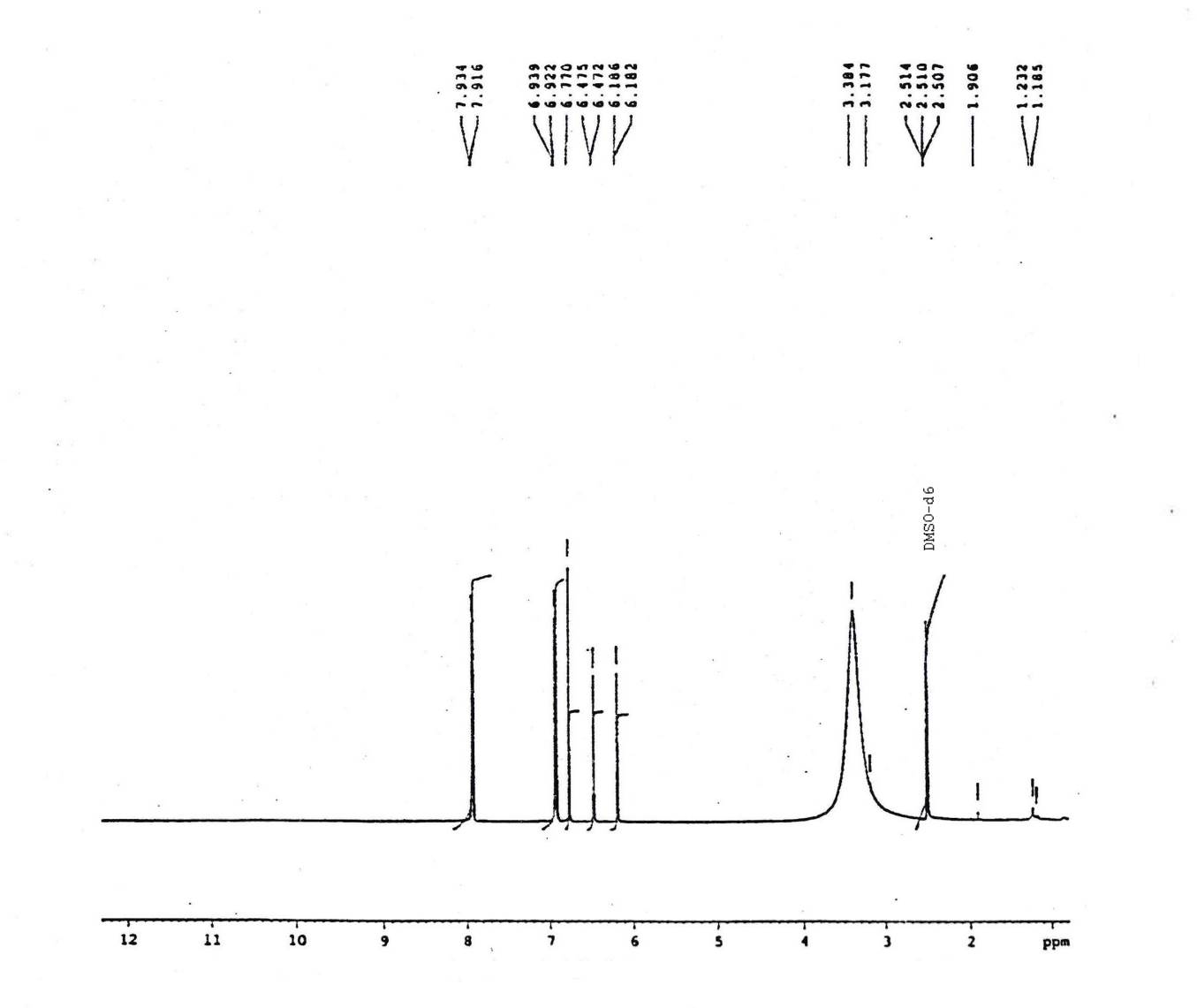


Fig. S1 ^1^H NMR (400 MHz, DMSO*-d*_6_) spectrum of compound **1**.


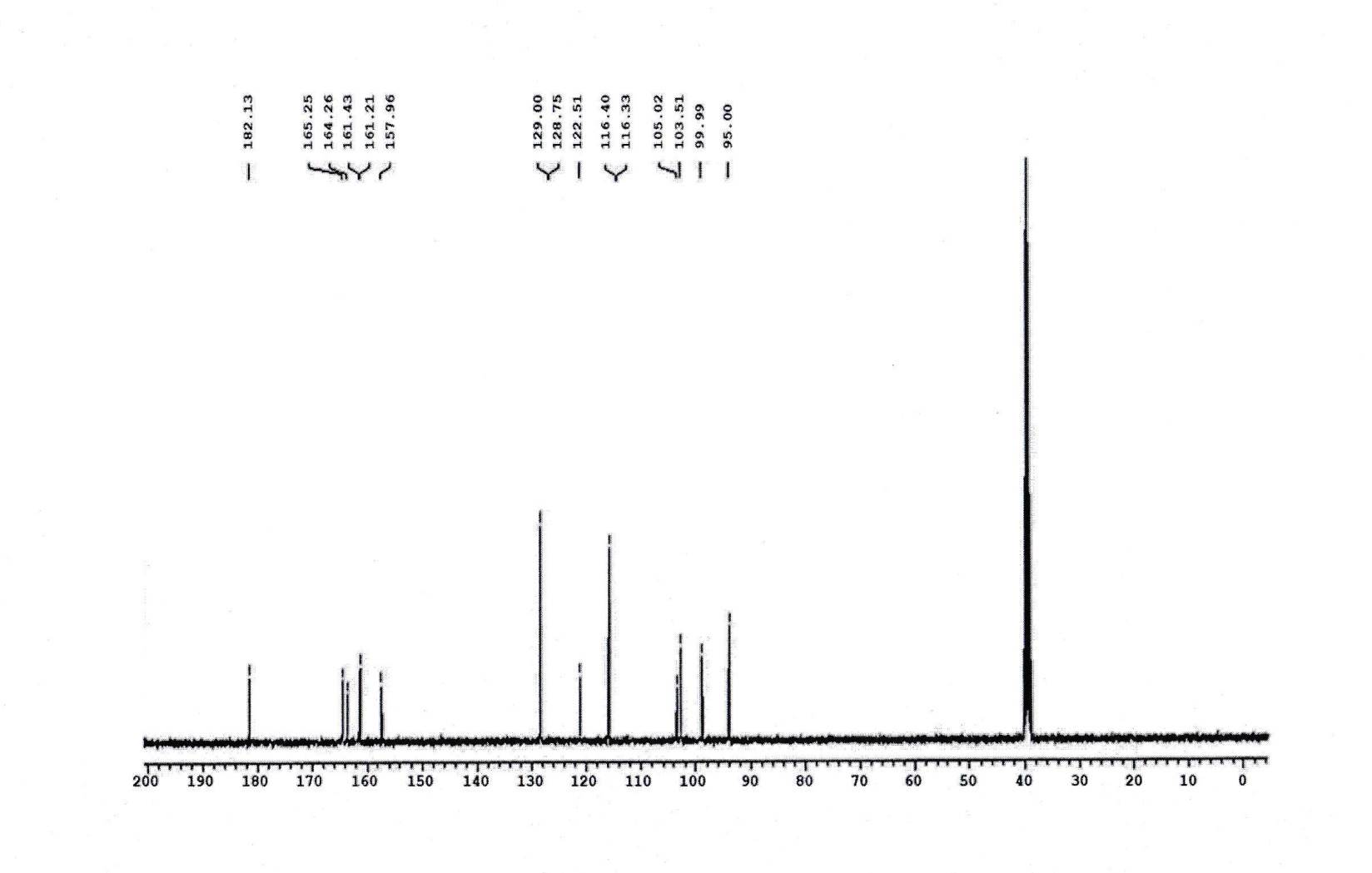


Fig. S2 ^13^C NMR (100 MHz, DMSO-*d*_6_) spectrum of compound **1**.
